# Analysis of cesarean section rates using Robson ten group classification system in a tertiary teaching hospital, Addis Ababa, Ethiopia: a cross-sectional study

**DOI:** 10.1186/s12884-020-03474-x

**Published:** 2020-12-09

**Authors:** Ferid A. Abubeker, Biruck Gashawbeza, Thomas Mekuria Gebre, Mekitie Wondafrash, Alula M. Teklu, Demis Degu, Delayehu Bekele

**Affiliations:** 1 Department of Obstetrics and Gynecology, Saint Paul’s Hospital Millennium Medical College, Addis Ababa, Ethiopia; 2 MERQ Consultancy PLC, Addis Ababa, Ethiopia

**Keywords:** Cesarean section, Robson classification, Vaginal delivery, Tertiary level facility, Low income countries

## Abstract

**Background:**

Cesarean section (CS) is an important indicator of access to, and quality of maternal health services. The World Health Organization recommends the Robson ten group classification system as a global standard for assessing, monitoring and comparing CS rates at all levels. This study aimed to assess the rate of CS and perform an analysis based on Robson classification system.

**Methods:**

A facility-based cross-sectional study was conducted at a tertiary hospital in Addis Ababa, Ethiopia. Data were collected from medical charts of all women who delivered from January-June 2018. The overall CS rate was calculated then women were categorized into one of the ten Robson groups. Relative size of each group, contribution of each group to the overall CS rate, and CS rate within each group were calculated.

**Results:**

A total of 4,200 deliveries were analyzed. Of these 1,459 (34.7%) were CS. The largest contributors to the overall CS rate were Group 10 (19.1%), Group 2 (18.3%), Group 5 (17.1%), and Group 4 (15.8%). There was also a high rate of pre-labor CS in Group 2, Group 4, and Group 10.

**Conclusion:**

Through implementation of the Robson ten group classification system, we identified the contribution of each group to the overall CS rate as well as the CS rate within each group. Group 10 was the leading contributor to the overall CS rate. This study also revealed a high rate of CS among low-risk groups. These target groups require more in-depth analysis to identify possible modifiable factors and to apply specific interventions to reduce the CS rate. Evaluation of existing management protocols and further studies into indications of CS and outcomes are needed to design tailored strategies and improve outcomes.

## Background

In 1985 the World Health Organization (WHO) stated a cesarean section (CS) rate higher than 10–15% is not justified for any region [1]. Thirty years after the publication of the WHO guidelines, there is no consensus about the optimal CS rate and appropriate interpretation of this indicator remains a topic of debate. More recent efforts to determine the optimal cs rate also had limitations due to lack of external validity and confounders [2, 3].

Despite its limitations, the proportion of cesarean section (CS) at a population level is an important indicator of access to, and quality of maternal health services offered in a country [4]. In 2016 the national population-based CS rare for Ethiopia was about 3%, a figure far below the WHO optimum range of 10–15%. The sub-national regional figures varied widely ranging from 25% in the capital, Addis Ababa, to less than 1% in more rural regions. Furthermore, the overall CS rates differ significantly between different institutions [5].

Several reasons can explain variations in institutional rates of CS. These include the inherent differences in patient characteristics, type of institution and available resources. In addition, institutional differences in obstetric practice and pregnancy and labor management protocols can account for this variation [6]. Therefore, population-based CS rates should not be considered as recommended targets at facility level. Indeed, systems designed to monitor cesarean section rates at facilities should take into account these differences. CS rates should no longer be thought of being too high or too low but rather whether or not they are appropriate. Thus, CS should only be conducted based on medical indications, and efforts should be directed towards improving access to all women in need rather than striving to achieve an arbitrary rate [6, 7].

To this end, policymakers, program managers, clinicians, and administrators need a standardized and internationally accepted classification system to monitor and compare CS rates in a meaningful, reliable and action-oriented manner [8]. A systematic review of existing CS classification system conducted in 2011 identified 27 different classification systems of which Robson’s Ten Group Classification System (RTGCS) was found to be the best option [9].

The Robson classification system classifies all deliveries into ten mutually exclusive and totally inclusive groups based on a set of predefined obstetric parameters. These include parity, previous CS, onset of labor, fetal presentation, number of fetuses and gestational age (Table 1). Each Robson group is further analyzed to assess its relative size to the obstetric population, its contribution to the overall CS rate, and the CS rate within the group [8]. The classification system is simple to use and enables auditing and analyzing CS rates as it is based on routinely documented obstetrics characteristics of individual woman without relying on the indication for CS.

Effective application of the RTGCS offers several advantages. It allows identification of the Robson groups that make significant contributions to the overall CS rate. This is a crucial step in the audit process as interventions that make even small changes to the CS rate within these target groups can bring about significant changes to the overall CS rate [7, 8]. The system is easily reproducible and offers a standardized comparison method within a particular institution over time or between institutions at a national, regional or global level [6–9]. In addition, RTGCS can inform the impact of interventions at both institutional and national levels by providing a benchmark and analyzing the trends in the overall and group-specific CS rates over time [10].

Cognizant of its advantages and simplicity, the WHO and the International Federation of Gynecology and Obstetrics (FIGO) recommend the Robson classification system as a global standard for assessing, monitoring and comparing CS rates among nations and within institution over time, and between institutions, regardless of their level of complexity [6, 11, 12].

**Table 1:** Robson ten group delivery classification system

Groups	Description
Group 1	Nulliparous, single cephalic, ≥ 37 weeks, in spontaneous labor
Group 2	Nulliparous, single cephalic, ≥ 37 weeks, induced or CS before labor
	2a- Nulliparous, singleton, cephalic, ≥ 37 weeks’ gestation, induced labor.
	2b- Nulliparous, singleton, cephalic, ≥ 37 weeks’ gestation, cesarean section before labor.
Group 3	Multiparous (excluding previous cesarean section), singleton, cephalic, ≥ 37 weeks’ gestation, in spontaneous labor.
Group 4	Multiparous without a previous uterine scar, with singleton, cephalic pregnancy, ≥ 37 weeks’ gestation, induced or cesarean section before labor.
	4a- Multiparous without a previous uterine scar, with singleton, cephalic pregnancy, ≥ 37 weeks’ gestation, induced labor.
	4b- Multiparous without a previous uterine scar, with singleton, cephalic pregnancy, ≥ 37 weeks’ gestation, cesarean section before labor.
Group 5	Previous cesarean section, singleton, cephalic, ≥ 37 weeks’ gestation.
Group 6	All nulliparous with a single breech.
Group 7	All multiparous with a single breech (including previous cesarean section).
Group 8	All multiple pregnancies (including previous cesarean section).
Group 9	All women with a single pregnancy in transverse or oblique lie (including those with previous cesarean section).
Group 10	All singleton, cephalic, < 37 weeks’ gestation pregnancies (including previous cesarean section).

Many obstetric units around the world have successfully utilized RTGCS. Application of the classification system in different institutions across the world have yielded similar results, although some had significant differences [13–16]. The aim of this study was thus to assess the rate of CS in our institution and perform an analysis based on Robson ten group classification system.

## Methods

### Study design, setting, and participants

This was a cross-sectional study conducted at Saint Paul’s Hospital Millennium Medical College (SPHMMC) in Addis Ababa, Ethiopia. SPHMMC is a tertiary care facility conducting close to 10,000 deliveries per annum. It is also a public teaching hospital and mainly serves as a referral center for high-risk cases.

The study population included all women who gave birth from January to June 2018. We excluded laparotomy done for uterine rupture and deliveries before fetal viability. In the Ethiopian context, viability is considered after gestational age of 28 weeks or birth weight ≥ 1,000 g, if gestational age is unknown [17].

### Data source and variables

Data were collected by trained data collectors using a structured data extraction template on Open Data Kit for an android platform and saved on a central server. Medical charts were reviewed to collect relevant obstetric information. This includes past obstetric history (parity and previous CS), onset of labor (spontaneous, induced, or CS before labor), fetal presentation or lie (cephalic, breech, transverse or oblique), number of fetuses (single or multiple), mode of delivery (vaginal or CS), and gestational age (term or preterm). Gestational age was calculated either from menstrual date or obstetric ultrasound done before 24 weeks of pregnancy. For cases with no gestational age milestone, we used birth weight as a proxy indicator of gestational age. Birth weight < 2,500 g was considered preterm and birth weight ≥ 2,500 g was considered term [18]. This strategy has been employed in other studies conducted in similar settings [14, 19, 20].

### Data processing and analysis

Data was exported to and analyzed using IBM SPSS Statistics for Windows, version 20 (IBM Corp., Armonk, N.Y., USA). The overall CS rate at the institution was calculated first. We coded all abstracted data and women were categorized into one of the ten Robson groups. For each group, size relative to the entire obstetric population, contribution to the overall CS rate, and CS rate within the group were calculated.

## Results

Over the six-month period, a total of 4,226 women presented for labor and delivery. Five patients were excluded for uterine rupture and 21 were excluded with pre-viable deliveries. Thus, a total of 4,200 deliveries were analyzed. The mean age of participants was 26.4 years (SD 4.7). The rate of CS was 34.7% (Table 2).

**Table 2:** Characteristics of women who gave birth at SPHMMC, Addis Ababa, Ethiopia, January-June 2018

Characteristics	Frequency (n)	Percentage (%)
**Age (years)**		
< 20	174	4.2
20–34	3,719	88.5
≥ 35	307	7.3
**Parity**		
0	1,980	47.1
1–4	2,147	51.1
≥ 5	73	1.8
**Gestational Age**^a^		
< 37weeks	821	19.5
≥ 37 weeks	3,379	80.5
**Onset of labor**		
Spontaneous	2,787	66.4
Induced	346	8.2
CS before onset of labor	1,067	25.4
**Fetal presentation/lie**		
Cephalic	3,967	94.5
Breech	219	5.2
Transverse/Oblique	14	0.3
**Number of fetus**		
Single	4,024	95.8
Multiple	176	4.2
**Mode of delivery**		
Vaginal delivery	2,741	65.3
Cesarean section	1,459	34.7
**Mode of operation**		
Emergency	935	64.1
Scheduled	524	35.9
**Fetal outcome**^b^		
Live birth	4,138	94.5
Stillbirth	240	5.5
**Birth weight (gram)**^b^		
< 1,000	30	0.7
1,000–2,499	953	21.8
2,500-3,999	3,234	73.8
≥ 4,000	161	3.7

^a^For 839 women with unknown gestational age, birth weight was used as a proxy indicator, 177 newborns had birth weight < 2,500 g and were considered < 37 weeks whereas 662 newborns had birth weight ≥ 2,500 g and were considered ≥ 37 weeks

^b^Rate calculated per total number of babies born

Women in Group 1 (nulliparous women with single cephalic pregnancy at term in spontaneous labor) made the largest contribution to the obstetric population accounting for 26.7% of all deliveries. This was followed by Group 3 (multiparous women with single cephalic pregnancy at term in spontaneous labor without previous CS) and Group 10 (all women with single cephalic pregnancy before term, including those with previous CS) which accounted for 22.2% and 15.9% respectively. Group 5 (all multiparous women with single cephalic pregnancy at term and at least one previous uterine scar) was the fourth largest group accounting for 9.5% of the obstetric population (Table 3).

The largest contributors to the overall CS rate were Group 10 (19.1%), Group 2 (nulliparous women with single cephalic pregnancy at term who either had an induction of labor or CS before the onset of labor) (18.3%), Group 5 (17.1%), and Group 4 (multiparous women with single cephalic pregnancy at term without previous CS who either had an induction of labor or CS before the onset of labor) (15.8%). These 4 groups contributed for about 70% of all cesarean deliveries (Table 3).

**Table 3:** Proportion of each Robson groups, CS rate in each group, and their relative and absolute contribution to overall CS rate at SPHMMC, Addis Ababa, Ethiopia, January-June 2018

Robson Group	Number of CS in group	Total number of women in group	Group size (%)^a^	Group CS rate (%)^b^	Absolute group contribution to overall CS rate (%)^c^	Relative group contribution to overall CS rate (%)^d^
Group 1	156	1,121	26.7	13.9	3.7	10.7
Group 2	267	368	8.8	72.6	6.4	18.3
2a	34	135	3.2	25.2	0.8	2.3
2b	233	233	5.6	100.0	5.6	16.0
Group 3	66	934	22.2	7.1	1.6	4.5
Group 4	231	327	7.8	70.6	5.5	15.8
4a	6	102	2.4	5.9	0.1	0.4
4b	225	225	5.4	100.0	5.4	15.4
Group 5	249	397	9.5	62.7	5.9	17.1
Group 6	48	94	2.2	51.1	1.1	3.3
Group 7	56	101	2.4	55.4	1.3	3.8
Group 8	95	176	4.2	54.0	2.3	6.5
Group 9	13	13	0.3	100.0	0.3	0.9
Group 10	278	669	15.9	41.6	6.6	19.1
**Total**	**1,459**	**4,200**	**100**	**34.7**	**34.7**	**100**

^a^Group size (%) = n of women in the group/total N women delivered in the hospital × 100

^b^Group CS rate (%) = n of CS in the group/total N of women in the group × 100

^c^Absolute group contribution (%) = n of CS in the group/total N of women delivered in the hospital × 100

^d^Relative group contribution (%) = n of CS in the group/total N of CS in the hospital × 100

Further subgroup analysis of women in Group 10 showed 323 (48.3%) had spontaneous onset of labor while 107 (16%) were induced. The remaining 239 (35.7%) underwent CS before the onset of labor. A similar analysis showed 233 (63.3%) in Group 2 and 225 (68.8%) in Group 4 had pre-labor CS.

Group 3 accounted for 4.5% of the overall CS rate and 7.1% of women within this group had CS. Group 6 and Group 7 represent nulliparous and multiparous women with breech presentation respectively. Together these two groups contributed 7.1% to the overall CS whereas the CS rate within each group was about 50% (Table 3).

## Discussion

Cesarean section is a key intervention to decrease maternal and neonatal morbidity and mortality. It is also one of the best indicators of the quality of maternal health services [4]. Despite its proven benefits, it has associated complications such as infection, bleeding, anesthetic accidents and even death. Future pregnancies can also be complicated by spontaneous preterm birth, uterine rupture, and abnormal placentation. These risks are higher for women in resource-limited settings with poor access to comprehensive obstetric care [6, 21].

Thus, to optimize outcomes, facilities should initiate a detailed and rigorous assessment of their practice vis-à-vis the case mix of obstetric population they serve. The Robson ten group classification system enables institution-specific monitoring and auditing and can be a powerful tool to inform practice across different settings [6, 8].

In this study, we implemented the RTGCS and assessed the proportion of each group in the obstetric population, the contribution of CS in each group to the overall CS rate and the CS rate within each group.

In our study, Group 1 and Group 3 represented the two largest groups presenting for labor and delivery. This finding is consistent with a study done in India where Group 1 and Group 3 contributed to 24.2% and 19.4% of all deliveries respectively [16]. Similarly, studies done in Brazil, Italy, and Tanzania showed Group 3 and Group 1 were the two most represented obstetric groups [13–15].

Group 10 was found to be the third-largest obstetric group. The contribution of this group to the overall CS rate depends on its size [7]. As such, Group 10 made the highest contribution to the CS rate accounting for nearly one in five CS deliveries. This is in sharp contrast with a study done at a university hospital in Eastern Ethiopia, where Group 10 was the 6th place contributor to the overall CS rate, accounting for 6% of CS deliveries [19]. This variation can be explained by the significant difference in the obstetric population served by the two facilities. Our study was done in a tertiary referral hospital with a dedicated maternal-fetal medicine unit. A significant proportion of care is given to mothers with major obstetric and medical comorbidities who may require interventions, increasing the likelihood of iatrogenic prematurity. This can account for the higher proportion of Group 10 and its contribution to the CS rate in our setting. In fact, our finding is consistent with studies conducted in other tertiary care facilities [7, 8]. A study from a tertiary unit in Italy also showed Group 10 was the second largest contributor to the CS rate [15].

About 20% of women in our study did not have a milestone to ascertain gestational age. This is not unusual as the national guideline does not include routine dating ultrasound during antenatal care follow up [22] and has not yet adopted the WHO recommendation of at least one ultrasound scan before 24 weeks of gestation [23]. In this study, birth weight was used as an indirect estimate of gestational age for those women we could not ascertain gestational age. This adaptation has also been used in other studies that implemented the RTGCS in low-resource settings. For example, Tura AK et al. [19] and Schantz C et al. [24] used birth weight of ≥ 2,500 g as proxy to term when data on gestational age was not available whereas other studies applied the method to their entire dataset and defined gestational age solely based on birth weight [14, 20, 25].

Using neonatal birth weight as a proxy indicator of gestational age can however result in misclassification of growth-restricted newborns as preterm and potentially increase the relative proportion of Group 10. To test for this, we performed a separate analysis after excluding the 839 women with unknown gestational age. However, there was no change to the relative proportion of Group 10 and its contribution to the CS rate, and the group remained the third largest group and the leading contributor to the overall CS rate.

In addition, the CS rate within Group 10 was found to be 41%, which indicates the possibility of a high rate of pre-labor CS (Table 3) [7]. Subgroup analysis shows one-third of women in this group underwent CS before the onset of labor. Further examination of the indications for CS can help us understand and design tailored interventions to reduce the CS rate in this group. This is especially relevant as interventions to reduce the CS rate within this particular group were found to be successful in other facilities [13].

Group 2 and Group 4 were also important contributors to the overall CS rate, accounting for one-third of CS deliveries. The CS rate within each group was also about 70%. Existing evidence suggests a high pre-labor CS rate at a particular institution if the CS rate within Group 2 and Group 4 is more than 35% and 20%, respectively [7]. Subdividing these groups into induced labor and CS before labor provides useful information regarding the proportion of pre-labor CS and the success of induction (Table 3). This is particularly important as women in these groups are considered low risk. Our subgroup analysis showed a large proportion of women in both groups underwent pre-labor CS. This calls for further investigation of the indications for pre-labor CS. Similarly, a high rate of CS in these low risk groups was observed in high resource settings like Italy, Singapore and Brazil [15, 26, 27]. For instance, the CS rate within Group 2 and Group 4 was 82% and 62% respectively at a public hospital in Brazil [27].

Several studies across different settings identified Group 5 as the leading contributor to the CS rate [13, 14, 16]. In our study, Group 5 was the third-largest contributor to the overall CS rate and its relative size to the obstetric population was less than 10%. These findings are suggestive of relatively low CS rate in the previous years [7]. Indeed, in 2016 the national institutional and population-based CS rates were 4% and 2.7% respectively [5].

Though the safety and long-term benefits of vaginal birth after cesarean (VBAC) are well established, 62% of women in Group 5 underwent repeat CS (Table 3). Thus, there is a need to evaluate the proportion of women who were offered a trial of labor and the success rate of VBAC. This will enable the design and implementation of antenatal counseling strategies and labor management protocols, reducing the number of repeat CS.

The contribution of Group 3 to the overall CS rate was small. However, the high rate of CS within this group is an alarming finding. The group represents low-risk women and the CS rate within this group is not expected to be higher than 3%. Auditing this group is an effective means to assess how an institution manages labor [7]. Thus, evaluation of labor management protocols in our institution is warranted.

Since the publication of the term breech trial, there is a global shift towards CS among women with breech presentation [28, 29]. Consequently, several studies showed a high rate of CS in Group 6 and 7 [13, 15]. However, in our study, nearly half of breech presentations both in nulliparous and multiparous women were delivered vaginally (Table 3). This is similar to the 40% vaginal breech delivery observed in another teaching hospital in southwest Ethiopia [30]. A more liberal national and institutional protocols that allow assisted vaginal breech delivery in selected women can explain these observations [22]. Though we find this practice encouraging, further analysis should be done to assess maternal and perinatal outcomes among these groups.

The strengths of this study include the large sample size and availability of complete data for analysis. The results of this study can serve as baseline data to monitor trends of CS rate over time in our institution, as well as to compare our practice with that in other institutions.

This study also has some limitations. Our definition of fetal viability based on gestational age of 28 weeks or birth weight of ≥ 1,000 g may affect the rate of CS and the relative size of Robson’s groups. This in turn can impact the generalizability of our findings to other countries. Findings from RTGCS are only a starting point and should be viewed as a means, not an end. We now have a clear insight about “who” is having CS but not “why” the CS is being performed. Crucial variables such as indications, maternal and perinatal outcomes, are not incorporated, limiting the extent to which conclusions can be drawn from our study.

## Conclusions

We used the RTGCS to identify specific groups that contributed the most to the overall CS in our setting. Group 10 was the leading contributor to the overall CS rate. This study also revealed a high rate of CS among low-risk groups. These target groups require more in-depth analysis to identify possible modifiable factors and to apply specific interventions to reduce the CS rate. Evaluation of existing management protocols and further studies into indications of CS and outcomes in our setting are needed to design tailored strategies and improve outcomes.

## Data Availability

All dataset used and/or analysed during the current study are available from the croosponding author on reasonable request.

## Abbreviations

CS: Cesarean Section
FIGO: International Federation of Gynecology and Obstetrics
RTGCS: Robson’s Ten Group Classification System
SPHMMC: Saint Paul’s Hospital Millennium Medical College
VBAC: vaginal birth after cesarean
WHO: World Health Organization
